# Viral Etiologies of Hospitalized Acute Lower Respiratory Infection Patients in China, 2009-2013

**DOI:** 10.1371/journal.pone.0099419

**Published:** 2014-06-19

**Authors:** Luzhao Feng, Zhongjie Li, Shiwen Zhao, Harish Nair, Shengjie Lai, Wenbo Xu, Mengfeng Li, Jianguo Wu, Lili Ren, Wei Liu, Zhenghong Yuan, Yu Chen, Xinhua Wang, Zhuo Zhao, Honglong Zhang, Fu Li, Xianfei Ye, Sa Li, Daniel Feikin, Hongjie Yu, Weizhong Yang

**Affiliations:** 1 Division of Infectious Disease, Key Laboratory of Surveillance and Early-warning on Infectious Disease, Chinese Centre for Disease Control and Prevention, Beijing, China; 2 Yunnan Provincial Center for Disease Control and Prevention, Kunming, China; 3 Centre for Population Health Sciences, Global Health Academy, The University of Edinburgh, Edinburgh, United Kingdom; 4 Public Health Foundation of India, New Delhi, India; 5 National Institute for Viral Disease Control and Prevention, Chinese Center for Disease Control and Prevention, Beijing, China; 6 Key Laboratory of Tropical Disease Control, Ministry of Education, Sun Yat-Sen University, Guangzhou, China; 7 State Key Laboratory of Virology, College of Life Sciences, Wuhan University, Wuhan, China; 8 Institute of Pathogen Biology, Chinese Academy of Medical Sciences & Peking Union Medical College, Beijing, China; 9 Beijing Institute of Microbiology and Epidemiology, State Key Laboratory of Pathogen and Biosecurity, Beijing, China; 10 Shanghai Public Health Clinical Center, Shanghai, China; 11 State Key Laboratory for Diagnosis and Treatment of Infectious Diseases, First Affiliated Hospital, School of Medicine, Zhejiang University, Hangzhou, China; 12 Gansu Provincial Center for Disease Control and Prevention, Lanzhou, China; 13 Liaoning Provincial Center for Disease Control and Prevention, Shenyang, China; 14 Division of Viral Diseases, National Center for Immunization and Respiratory Diseases, Centers for Disease Control and Prevention, Atlanta, Georgia, United States of America; Kliniken der Stadt Köln gGmbH, Germany

## Abstract

**Background:**

Acute lower respiratory infections (ALRIs) are an important cause of acute illnesses and mortality worldwide and in China. However, a large-scale study on the prevalence of viral infections across multiple provinces and seasons has not been previously reported from China. Here, we aimed to identify the viral etiologies associated with ALRIs from 22 Chinese provinces.

**Methods and Findings:**

Active surveillance for hospitalized ALRI patients in 108 sentinel hospitals in 24 provinces of China was conducted from January 2009-September 2013. We enrolled hospitalized all-age patients with ALRI, and collected respiratory specimens, blood or serum collected for diagnostic testing for respiratory syncytial virus (RSV), human influenza virus, adenoviruses (ADV), human parainfluenza virus (PIV), human metapneumovirus (hMPV), human coronavirus (hCoV) and human bocavirus (hBoV).

We included 28,369 ALRI patients from 81 (of the 108) sentinel hospitals in 22 (of the 24) provinces, and 10,387 (36.6%) were positive for at least one etiology. The most frequently detected virus was RSV (9.9%), followed by influenza (6.6%), PIV (4.8%), ADV (3.4%), hBoV (1.9), hMPV (1.5%) and hCoV (1.4%). Co-detections were found in 7.2% of patients. RSV was the most common etiology (17.0%) in young children aged <2 years. Influenza viruses were the main cause of the ALRIs in adults and elderly. PIV, hBoV, hMPV and ADV infections were more frequent in children, while hCoV infection was distributed evenly in all-age. There were clear seasonal peaks for RSV, influenza, PIV, hBoV and hMPV infections.

**Conclusions:**

Our findings could serve as robust evidence for public health authorities in drawing up further plans to prevent and control ALRIs associated with viral pathogens. RSV is common in young children and prevention measures could have large public health impact. Influenza was most common in adults and influenza vaccination should be implemented on a wider scale in China.

## Introduction

Acute lower respiratory infections (ALRIs) continue to be an important cause of acute illnesses and mortality worldwide (especially in infants and young children) [1]–[4]. The Global Burden of Diseases, Injuries, and Risk Factors Study 2010 (GBD 2010) estimates that there were 2.8 million deaths due to lower respiratory infections globally in 2010 (5.3% of the total deaths) [4]. The incidence of ALRIs in children aged less than 5 years is estimated to be 0.22 episodes per child-year, with 11.5% cases progressing to severe episodes in low- and mid-income countries in 2010, and most cases occur in India, China, Pakistan, Bangladesh, Indonesia and Nigeria [5], [6]. The main etiological agents responsible for ALRIs include bacteria (*Streptococcus pneumoniae, Haemophilus influenzae* type b, Staphylococcus aureus, etc.), viruses, and fungi. Respiratory syncytial virus (RSV), human influenza viruses, human parainfluenza viruses type 1, 2, and 3 (PIV-1, PIV-2 and PIV-3), human rhinoviruses (HRV), adenoviruses (ADV), human metapneumovirus (hMPV), human coronavirus (hCoV), and human bocavirus (hBoV) have been identified among patients with ALRI [7]–[10]. In China, ALRIs were the most frequent cause of child mortality in 2008, despite an astonishing 66% reduction in pneumonia-specific mortality rates between 2002–2007 (from about 9·4 to 3·2 per 1000 live births) [11]. Moreover, most pneumonia deaths occur in poor rural communities.

The majority of the studies contributing data on the epidemiology of the etiologic agents of ALRIs are from industrialized countries. Although several studies have reported the prevalence of viral infections in outpatients and inpatients in local areas of China [12]–[17], a large-scale study across multiple provinces and seasons has not been previously reported. We aimed to identify the viral etiologies associated with ALRIs from patients across 22 provinces of China between 2009–2013.

## Methods

### Ethics Statement

The China’s Ministry of Health decided that since data from patients with ALRI was part of continuing public health surveillance and implemented national surveillance guidelines; parents/guardians of participants in this study were only required to provide brief verbal consent during their enrollment, which was recorded in each questionnaire by their physicians. This project and the above procedure for obtaining consent were approved by the ethical review committee of the Chinese Center for Disease Control and Prevention (China CDC, Beijing, China).

### Setting and Patients Enrollment

In 2009, we initiated active surveillance for hospitalized ALRI patients in 108 sentinel hospitals in 24 provinces of China. The sentinel sites were chosen after carefully considering capacities of surveillance and laboratory testing, and geographical representativeness. A national surveillance protocol including guidelines for patient enrollment, specimen collection and laboratory testing, case reporting form and protocols for data transmission and related standard operating procedures (SOP) in Chinese language were developed by Chinese Center for Disease Control and Prevention (China CDC, Beijing) and the regional reference laboratories. This protocol was then used by all the participating hospitals and laboratories [18].

Patients admitted to the wards or intensive care unit (ICU) of departments of internal medicine, pediatrics or infectious diseases in each of these sentinel hospital were screened by nurses and physicians for ALRI. A patient was considered to be having ALRI if they had: (1) at least one of listed manifestation of acute infection: measured fever (≥38°C), abnormal white blood cell (WBC) differential, leukocytosis (a WBC count more than 10,000/µL) or leukopenia (a WBC count less than 4,000/µL), and chill; (2) at least one of listed signs/symptoms of respiratory tract infection: cough, sputum, shortness of breath, lung auscultation abnormality (rale or wheeze), tachypnea, and chest pain. Among ALRI patients, those with a chest radiograph demonstrating punctate, patchy or uniform density opacity were defined as having radiographic evidence of pneumonia [17].

### Specimen Collection and Testing

Each week or month, the first 2–5 ALRI patients were enrolled for specimen collection in each sentinel hospital, based on their pre-defined target sample size. Cases were screened weekly in 49 hospitals and monthly in the other 59 hospitals. After hospital admission, physicians obtained verbal consent from eligible ALRI cases- or their parent/guardians, following which appropriate respiratory specimens (nasopharyngeal swab or aspirate, sputum, bronchoalveolar lavage or lung puncture aspirate), blood or serum were collected. Respiratory specimens were placed immediately in viral transport media (VTM) and stored at 4–8°C at the local hospital. Collected blood samples were placed at −20°C in vacuum blood tube on ice. Specimens were transferred to the 46 qualified laboratories (including 14 central, regional reference laboratories, and 32 local laboratories at prefecture or provincial level) for diagnostic testing. Most of the tests were completed within 24 hours after collection. At the laboratories, specimens in VTM were stored at −70°C if testing was not performed within 24 hours after collection.

**Figure 1:**
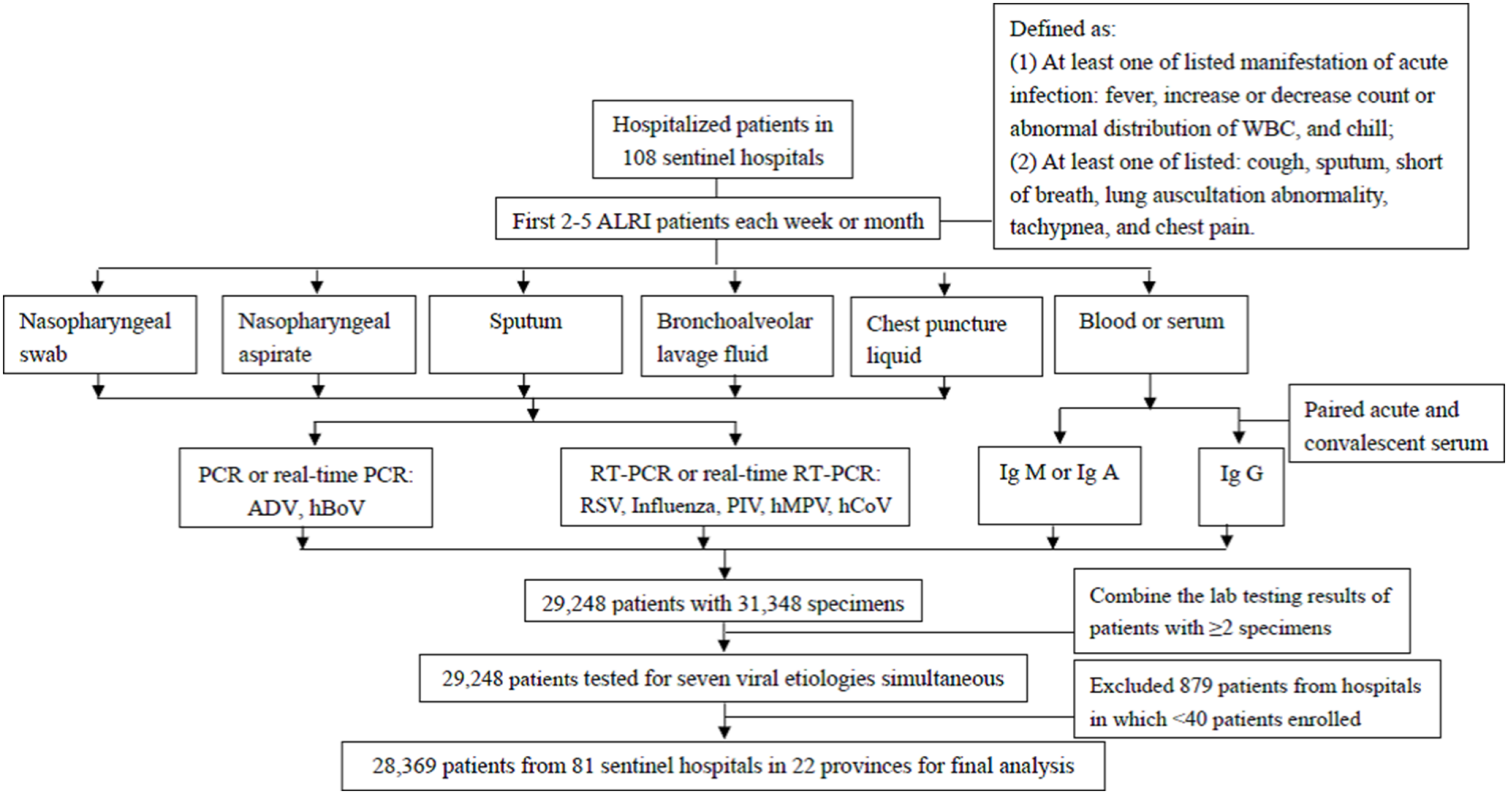
Enrollment of 28,369 hospitalized patients with acute lower respiratory infection tested for seven virus etiologies from January 1, 2009 to September 30, 2013 in China.

Each specimen was tested for the seven identified viral etiologies, i.e. RSV, human influenza virus, ADV, human parainfluenza virus (PIV), hMPV, hCoV and hBoV. The viral nucleic acid was directly extracted from respiratory specimens by using commercial kits (e.g. QIAmpMiniElute Virus Spin kit, QIAamp Viral RNA Mini kit or RNeasy Mini kit, by Qiagen, Valencia, CA) recommended by China CDC. Polymerase chain reaction (PCR) or real-time PCR were performed to detect ADV and hBoV, and reverse transcriptase- PCR (RT-PCR) or real-time RT-PCR were performed to detect the other five viral agents as described previously [13], [17]. Ig M or Ig A of the targeted viruses were detected in a single blood or serum specimen, and Ig G were detected in paired acute and convalescent sera, by enzyme-linked immunosorbent assay (ELISA) using recommended commercial indirect immunofluorescence kits (xTAG Respiratory Viral Panel) according to the manufacturer’s protocol (Figure 1). These assays were performed in biosafety level 2 facility laboratories. All the laboratories used the same protocol (see primers and sequence information in Table S1 and Table S2 in File S1) [18], which undergoes quality control assessment by the China CDC. If any one of the targeted viruses was detected in the specimens, the patient was considered to be positive for that viral etiology. If two or more specimens from one patient were tested and one of them tested positive, we considered the patient to be positive for the virus (Figure 1). The cases where only a single pathogen identified was labelled as mono-infection, and the cases where two or more viruses were detected was regarded as co-detection.

### Data Collection and Statistical Analysis

Detailed demographic and clinical data of cases and laboratory results of specimens were collected by staff of sentinel hospitals and laboratories through a standardized case reporting form, and entered on a weekly basis into an online data management system established by China CDC.

We used descriptive statistics to summarize the spectrum of viral etiologies by age group in hospitalized ALRIs patients, and to analyze their temporal trends using monthly data during the study period. Statistical analysis was performed with SPSS (v18.0, SPSS, Chicago, IL, USA). Two tailed Mann-Whitney tests were used for the comparison of median age between patients positive and patients negative for viruses. Fisher’s Least Significant Difference (L-S-D) tests were used for multiple comparisons of median age between patients positive for different viruses, and Chi-square tests were used to analyze the frequency data. P<0.05 was considered to be statistically significant.

## Results

### Characteristics of Hospitalized ALRI Patients

**Figure 2:**
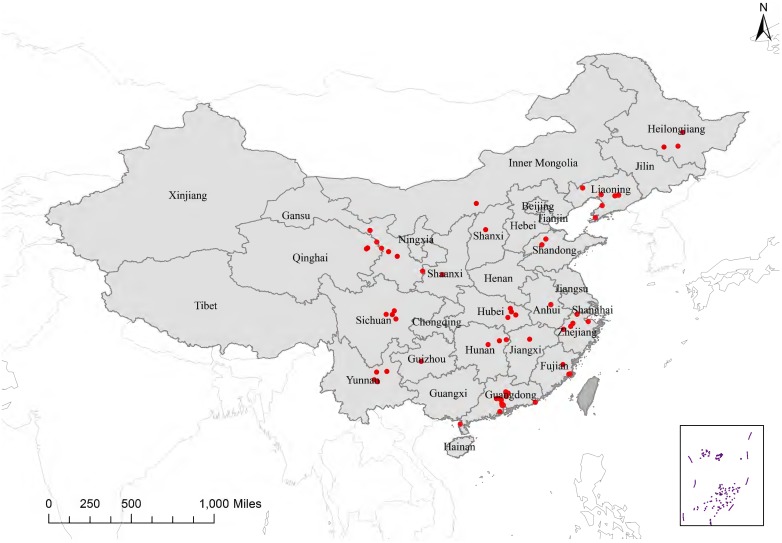
Location of 81 surveillance hospitals for hospitalized acute lower respiratory infection patients. The red dots indicate the location of the surveillance hospitals. A total of 81 hospitals in 22 provinces participate in acute lower respiratory infection surveillance for final analysis. The box indicates Spratly Islands in Southern China Sea.

During January 2009-September 2013, 29,248 hospitalized ALRIs patients contributing 31,348 specimens were enrolled from the 108 sentinel hospitals. Due to sparse patients (<40) in 27 sentinel hospitals, we just included 28,369 patients from 81 sentinel hospitals in 22 provinces who were tested for the aforementioned seven viral etiologies for final analysis (Figure 1 and Figure 2). Among the 28,369 ALRI patients, 60% were children aged <5 years and 12% were elderly aged ≥65 years, with a median age of 3 years [interquartile range (IQR), 0.7–24 years]. A temperature ≥38°C was documented in 43% of ALRI cases at the time of physical examination. Cough was the most common symptom (79%). Of 14,720 ALRI patients (52% of total) that had a chest X-ray performed, 10,115 (69%) were reported to have radiographic evidence of pneumonia (Table 1).

**Table 1:** Characteristics of enrolled hospitalized acute lower respiratory infection patients and laboratory-confirmed viral etiologies in China.

Characteristics	All ALRI patients (%)* [n = 28369]	Number (%)* of hospitalized ALRI patients with confirmed viral etiology									Negative [n = 17982]
		Any viral etiology [n = 10387]	RSV [n = 2795]	Influenza [n = 1869]	PIV [n = 1366]	ADV [n = 957]	hBoV [n = 551]	hMPV [n = 424]	hCoV [n = 393]	Co-detection [n = 2032]	
Male sex	18210 (64.2)	6899 (66.4)	1860 (66.5)	1231 (65.9)	898 (65.7)	618 (64.6)	358 (65)	268 (63.2)	263 (66.9)	1403 (69)	11311 (62.9)
Age, median (IQR^§^, years)	3 (0.7–24)	1 (0.5–4)	0.7 (0.3–2)	4 (1–32)	1 (0.4–3)	2 (1–5)	1 (0.5–2)	2 (0.8–4)	3 (0.9–24)	0.9 (0.4–2)	4 (1–47)
Age group											
<6 months	5298 (18.7)	2567 (24.7)	1086 (38.9)	171 (9.1)	358 (26.2)	109 (11.4)	126 (22.9)	62 (14.6)	62 (15.8)	593 (29.2)	2731 (15.2)
6–11 months	3333 (11.7)	1681 (16.2)	528 (18.9)	150 (8)	255 (18.7)	116 (12.1)	110 (20)	52 (12.3)	45 (11.5)	425 (20.9)	1652 (9.2)
12–23 months	3337 (11.8)	1627 (15.7)	422 (15.1)	199 (10.6)	202 (14.8)	168 (17.6)	137 (24.9)	69 (16.3)	47 (12)	383 (18.8)	1710 (9.5)
2–4 years	5159 (18.2)	2206 (21.2)	484 (17.3)	446 (23.9)	265 (19.4)	288 (30.1)	116 (21.1)	141 (33.3)	76 (19.3)	390 (19.2)	2953 (16.4)
5–9 years	2618 (9.2)	797 (7.7)	121 (4.3)	222 (11.9)	126 (9.2)	114 (11.9)	29 (5.3)	30 (7.1)	38 (9.7)	117 (5.8)	1821 (10.1)
10–14 years	892 (3.1)	209 (2)	29 (1)	56 (3)	27 (2)	30 (3.1)	7 (1.3)	6 (1.4)	18 (4.6)	36 (1.8)	683 (3.8)
15–49 years	2629 (9.3)	520 (5)	46 (1.6)	277 (14.8)	39 (2.9)	61 (6.4)	9 (1.6)	23 (5.4)	28 (7.1)	37 (1.8)	2109 (11.7)
50–64 years	1790 (6.3)	294 (2.8)	24 (0.9)	133 (7.1)	33 (2.4)	27 (2.8)	6 (1.1)	19 (4.5)	29 (7.4)	23 (1.1)	1496 (8.3)
≥65 years	3313 (11.7)	486 (4.7)	55 (2)	215 (11.5)	61 (4.5)	44 (4.6)	11 (2)	22 (5.2)	50 (12.7)	28 (1.4)	2827 (15.7)
Clinical history and physical examination											
T ≥38.0°C	11395/26542 (42.9)	3864/9695 (39.9)	833/2608 (31.9)	912/1784 (51.1)	431/1234 (34.9)	528/905 (58.3)	144/511 (28.2)	219/381 (57.5)	152/360 (42.2)	645/1912 (33.7)	7531/16847 (44.7)
Cough	22364 (78.8)	8816 (84.9)	2466 (88.2)	1567 (83.8)	1134 (83)	756 (79)	457 (82.9)	365 (86.1)	313 (79.6)	1758 (86.5)	13548 (75.3)
Runny nose	5350 (18.9)	2282 (22)	709 (25.4)	416 (22.3)	245 (17.9)	204 (21.3)	97 (17.6)	108 (25.5)	72 (18.3)	431 (21.2)	3068 (17.1)
Sore throat	3000 (10.6)	661 (6.4)	79 (2.8)	248 (13.3)	75 (5.5)	114 (11.9)	16 (2.9)	21 (5)	35 (8.9)	73 (3.6)	2339 (13)
Sputum production	12985 (45.8)	4863 (46.8)	1294 (46.3)	946 (50.6)	612 (44.8)	383 (40)	244 (44.3)	189 (44.6)	166 (42.2)	1029 (50.6)	8122 (45.2)
Chest pain	1345 (4.7)	218 (2.1)	23 (0.8)	77 (4.1)	35 (2.6)	27 (2.8)	11 (2)	5 (1.2)	20 (5.1)	20 (1)	1127 (6.3)
Tachypnea	1289 (4.5)	579 (5.6)	187 (6.7)	94 (5)	60 (4.4)	25 (2.6)	36 (6.5)	27 (6.4)	23 (5.9)	127 (6.3)	710 (3.9)
Difficulty breathing	3417 (12)	1261 (12.1)	393 (14.1)	224 (12)	146 (10.7)	80 (8.4)	68 (12.3)	52 (12.3)	36 (9.2)	262 (12.9)	2156 (12)
Headache	1691 (6)	397 (3.8)	60 (2.1)	144 (7.7)	36 (2.6)	70 (7.3)	17 (3.1)	12 (2.8)	14 (3.6)	44 (2.2)	1294 (7.2)
Fatigue	1963 (6.9)	408 (3.9)	53 (1.9)	179 (9.6)	53 (3.9)	46 (4.8)	7 (1.3)	15 (3.5)	22 (5.6)	33 (1.6)	1555 (8.6)
Abdominal pain	416 (1.5)	117 (1.1)	21 (0.8)	30 (1.6)	20 (1.5)	13 (1.4)	7 (1.3)	3 (0.7)	4 (1)	19 (0.9)	299 (1.7)
Diarrhea	1732 (6.1)	972 (9.4)	292 (10.4)	96 (5.1)	151 (11.1)	54 (5.6)	64 (11.6)	20 (4.7)	18 (4.6)	277 (13.6)	760 (4.2)
Lung rale sounds on auscultation	10190/22275 (45.7)	4720/8741 (54)	1501/2394 (62.7)	629/1492 (42.2)	581/1152 (50.4)	293/763 (38.4)	283/481 (58.8)	153/352 (43.5)	135/298 (45.3)	1145/1809 (63.3)	5470/13534 (40.4)
Radiographic evidence of pneumonia	10115/14720 (68.7)	4201/5876 (71.5)	1266/1713 (73.9)	647/984 (65.8)	526/763 (68.9)	296/433 (68.4)	246/348 (70.7)	193/230 (83.9)	109/171 (63.7)	918/1234 (74.4)	5914/8844 (66.9)

*Data is presented as no. (%) of patients unless otherwise indicated. Denominators for testing of fewer cases than full group are indicated. Percentages may not total 100 because of rounding. ^§^IQR: interquartile range.

### Characteristics of Patients with Laboratory Confirmed Viral Etiologies

Of 28,369 hospitalized ALRI patients tested for the seven viral etiologies, 10,387 (36.6%) were positive for at least one etiology. The median age of these patients was considerably lower than patients who were negative for any of the viruses (1 years vs 4 years) (P = 0.000, Mann-Whitney Test). Of the patients in who viruses were identified, those with confirmed influenza and hCoV had a higher median age (4 and 3 years) than patients positive for other viruses (0.7–2 years) (P = 0.000, L-S-D Test). Compared with patients testing positive for other viral etiologies, patients with laboratory confirmed influenza and hCoV more frequently complained of chest pain and fatigue. A similar pattern was observed in adults aged 50–64 years and the elderly (Table 1).

### Spectrum of Viral Agents

**Figure 3:**
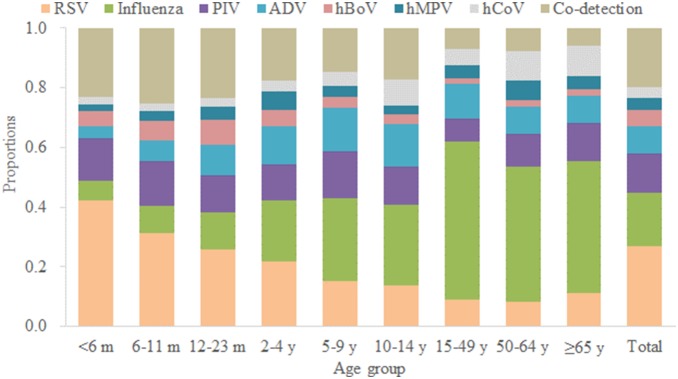
Average proportions of viral etiologies for hospitalized acute lower respiratory infection patients in 2009–2013 by age group.

**Figure 4:**
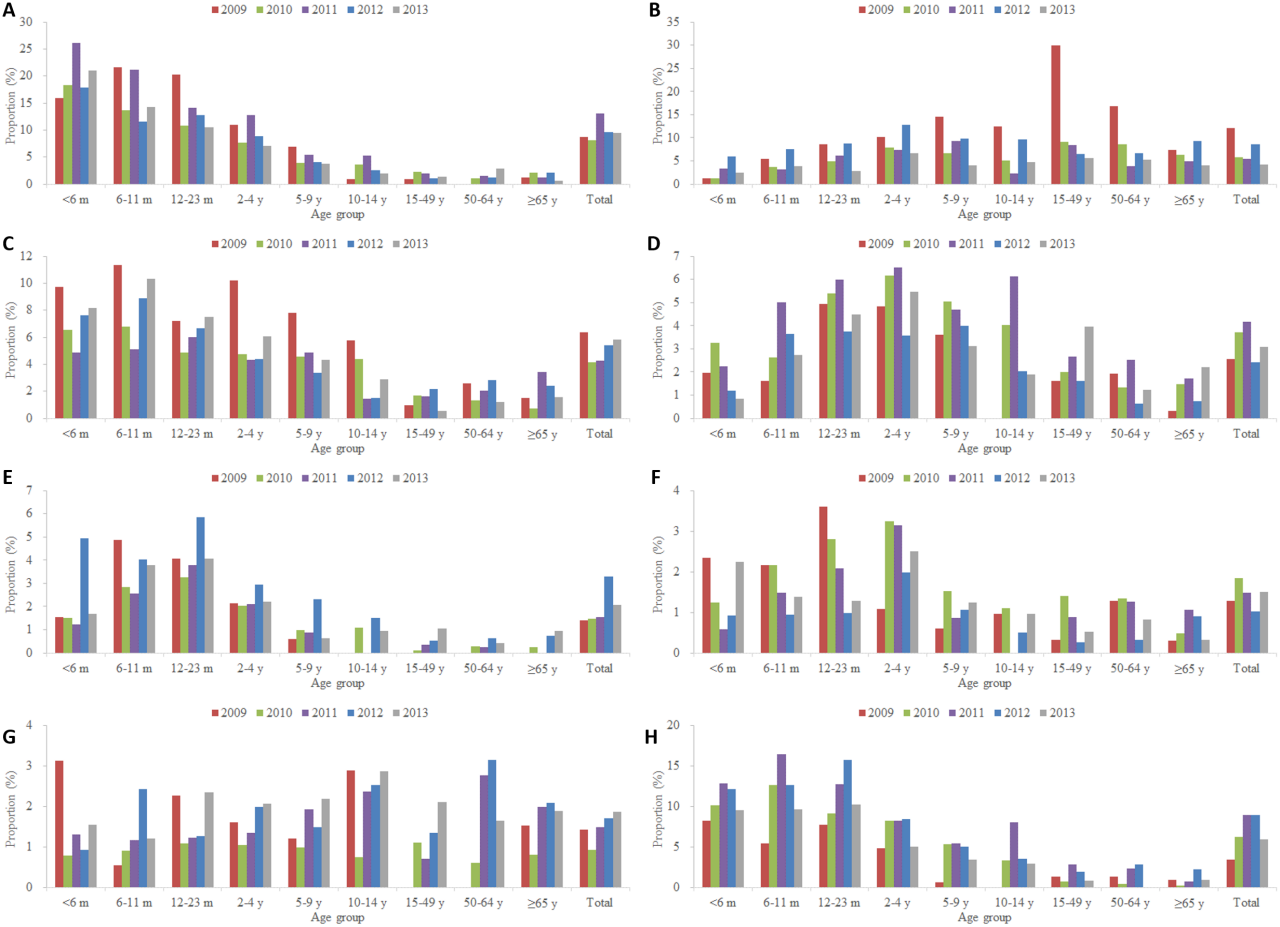
Proportion of viral etiologies for hospitalized acute lower respiratory infection patients by year. A) RSV. B) Influenza. C) PIV. D) ADV. E) hBoV. F) hMPV. Panel G) hCoV. H) Co-detection.

Of the 10,387 hospitalized ALRI patients confirmed with at least one viral etiology, the most frequent detected virus was RSV (in 2,795 patients, 9.9%), followed by influenza in 1,869 patients (6.6%), PIV in 1,366 patients (4.8%), ADV in 957 patients (3.4%), hBoV in 551 patients (1.9%), hMPV in 424 patients (1.5%) and hCoV in 393 patients (1.4%) (Table 1). Co-detections were found in 7.2% of patients. All the seven viral agents were detected in each age group (Figure 3). However, RSV was the most common etiology in very young children aged <2 years (17.0% of ALRI patients, Figure 4, Panel A). Influenza viruses were the mainly associated with ALRIs in adults (10.5% in 15–49 years) and elderly (7.4% in 50–64 years and 6.5% in ≥65 years) throughout the study period. During the A(H1N1)pdm09 pandemic, about 30% of the adult (15–49 years) ALRI admissions were associated with influenza. (Figure 4, Panel B). PIV, hBoV and hMPV infections were more frequent in young children (Figure 4, Panels C, E, F), ADV infections were more common in children aged 6 months-9 years (Figure 4, Panel D), while hCoV infection was evenly distributed in all-age groups (Figure 4, Panel G).

The proportion of respiratory viruses in ALRI patients has demonstrated substantial annual variation (P = 0.000, Chi-square test). This is more marked in case of influenza viruses, which had a much higher proportion in 2009 than that in the rest of the years (Figure 4, Panel B). A significant increase in hBoV proportion was observed in 2012 (Figure 4, Panel F) (P = 0.000, Chi-square test).

### Co-detection of Multiple Viral Etiologies

Among the 2032 ALRI patients with co-detection, two viruses were identified in 1709 cases (84.1% of cases with co-detection), three viruses were detected in 277 cases (13.6%), and four or more viruses were detected in 46 cases (Table 2). RSV was the most frequent etiology in cases with co-detection. RSV and another respiratory virus were detected in 1296 (53.8%) cases 354 cases positive in combination with influenza viruses and 272 cases with PIV. hBoV co-detection (with other viral pathogens) was observed in 413 (24.2%) of 1709 cases (75% of 551 cases with hBoV mono-infection).

**Table 2:** Co-detection of multiple viral etiologies in acute lower respiratory infections.

Viral etiologies	No. of cases		Viral etiologies	No. of cases
**Two pathogens**	**1709 (84.1%)**		FLU+RSV+hBoV	24
FLU+RSV	354		PIV+ADV+hBoV	16
RSV+PIV	272		RSV+ADV+hBoV	15
RSV+ADV	166		RSV+PIV+hCoV	15
RSV+hBoV	144		FLU+RSV+hCoV	11
PIV+hBoV	129		FLU+ADV+hBoV	9
FLU+PIV	99		FLU+PIV+hBoV	7
PIV+ADV	66		RSV+PIV+hMPV	7
RSV+hCoV	63		FLU+PIV+ADV	6
FLU+ADV	59		RSV+hMPV+hBoV	6
FLU+hBoV	55		PIV+ADV+hCoV	5
ADV+hBoV	51		RSV+ADV+hCoV	5
RSV+hMPV	43		ADV+hMPV+hBoV	4
FLU+hCoV	38		FLU+ADV+hCoV	3
PIV+hCoV	37		FLU+hMPV+hBoV	3
FLU+hMPV	26		PIV+ADV+hMPV	3
PIV+hMPV	26		PIV+hCoV+hBoV	3
hMPV+hBoV	18		PIV+hMPV+hBoV	3
ADV+hCoV	16		RSV+ADV+hMPV	3
hCoV+hBoV	16		FLU+hMPV+hCoV	2
hMPV+hCoV	16		FLU+PIV+hCoV	2
ADV+hMPV	15		RSV+hCoV+hBoV	2
**Three pathogens**	**277 (13.6%)**		ADV+hMPV+hCoV	1
FLU+RSV+PIV	39		FLU+RSV+hMPV	1
RSV+PIV+hBoV	29		RSV+hMPV+hCoV	1
RSV+PIV+ADV	27		**4 pathogens**	**43 (2.1%)**
FLU+RSV+ADV	25		**5 pathogens**	**3 (0.1%)**

### Temporal Trends of Viral Etiologies

**Figure 5:**
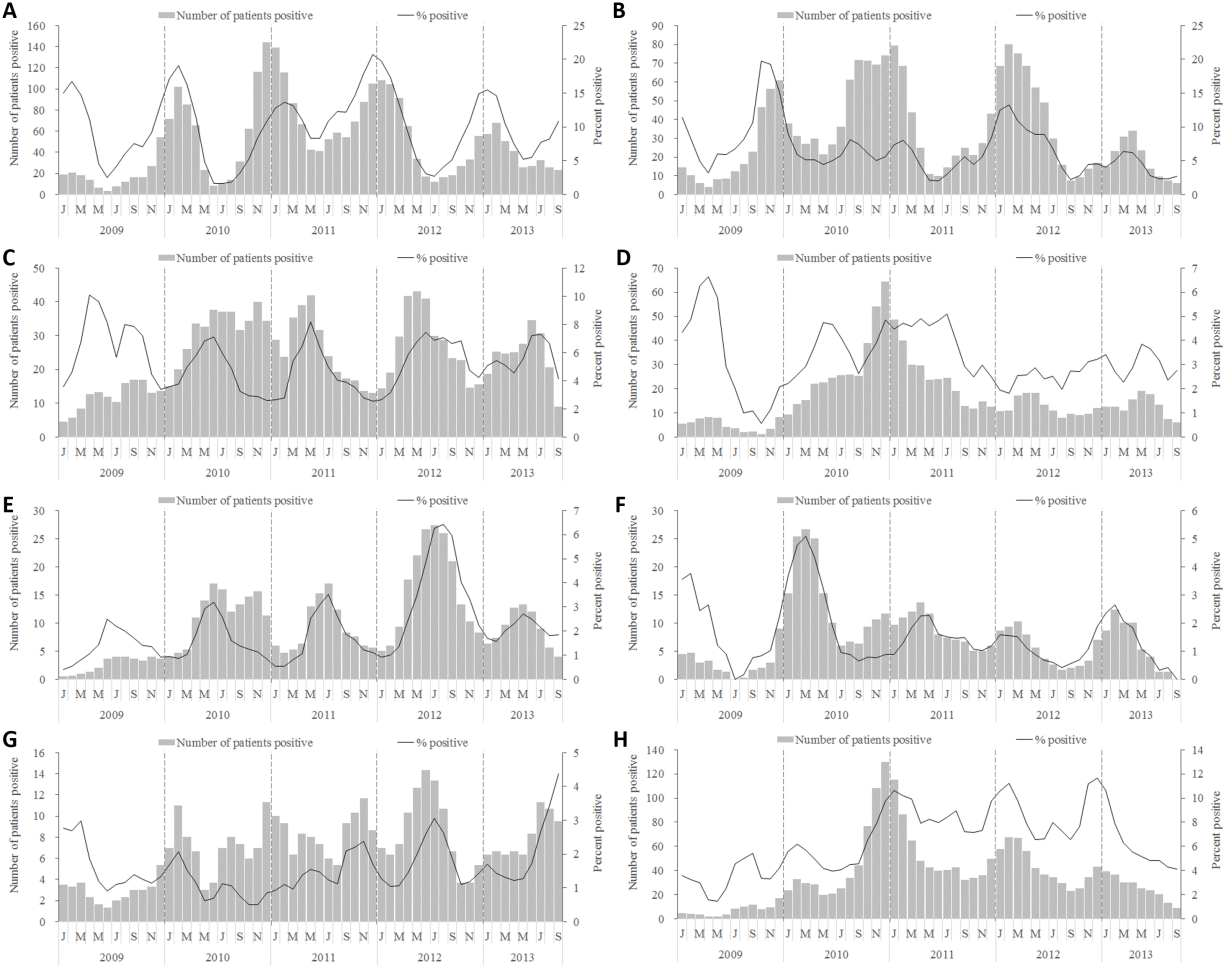
Number and percentage of patients positive by viral etiology. A) RSV. B) Influenza. C) PIV. D) ADV. E) hBoV. F) hMPV. G) hCoV. H) Co-detection.

Over the 57-month period, there were clear seasonal peaks for RSV, influenza, PIV, hBoV and hMPV infections. RSV activity was observed throughout the year during the 5 year period with an annual a peak in January–February each year (Figure 5, Panel A). Similarly Influenza circulation was observed throughout the year with peaks in autumn-winter. The peaks were higher in 2009 when A(H1N1)pdm09 influenza circulated in worldwide and Spring (January–March) in 2012, and relatively lower activity was observed in the post-pandemic seasons of 2010–2011 and 2013 (Figure 5, Panel B). PIV, hBoV and hMPV infections had a similar pattern of one peak annually, with peak PIV and hMPV infections observed in late spring (March–May) in most seasons and hBoV peaking in summer (June–July) (Figure 5, Panels C, E and F). There were no clear temporal trends for patients infected with ADV and hCoV (Figure 5, Panels D and G), even when analysis was stratified by age group (Figure S1 and Figure S2).

## Discussion

This study is the first to describe the viral etiologies in hospitalized ALRI patients using data from sentinel surveillance sites covering the majority of Chinese provinces over 5 consecutive years; and based on a standardized surveillance protocol and laboratory assays. A total of 28,369 hospitalized ALRI patients were enrolled from 2009–2013, and 36.6% were positive for at least one virus, which is consistent with published data reported from China and other countries [13], [18], [19].

Our findings that RSV was the leading pathogen identified in young children under two years hospitalized for lower respiratory tract infections demonstrates that RSV could also be associated with substantial morbidity and mortality in China, as reported in studies from other industrialized and developing countries [20]–[22]. This finding indicates that prevention strategies for RSV such immunization when a suitable vaccine is available in the future could have large public health impact in China. We also demonstrated that influenza viruses could lead to substantial burden on health care system especially in a large country like China with a rapidly aging population especially since influenza positivity rate was higher in adults and elderly. This is consistent with the reported estimates of influenza disease burden based on studies conducted in China and across the world [23]–[26]. However, influenza vaccination (which confers individual and herd immunity) has an extremely low coverage rate in China [27]. Widespread use of influenza vaccine should have a considerable impact on the influenza disease burden in China. PIV was the second most common pathogen identified in children aged less than 2 years. We also observed that ADV was an important virus associated with ALRI in children. These evidences will be important for China’s public health authorities to guide the priority for control of infectious diseases.

From a public health perspective, information on seasonality of pathogens is crucial to inform the timing of interventions, particularly for a climatically and economically diverse country as China [28]. China has an intensive national influenza surveillance network and influenza seasonality in different epidemiological regions was identified to be used as a basis to optimize the timing of future vaccination programs [29]. Our study demonstrates that although respiratory viruses circulate throughout the year, viruses like RSV, PIV, hBoV and hMPV have a clear seasonal trend. RSV activity peaked in January–February each year, and this is consistent with published reports from other studies in temperate regions where RSV occurred most frequently in the winter months [6].

Our study had a few limitations. Firstly, only viral pathogens were detected in our study; bacterial pathogens were not included, which prevented us from getting comprehensive data on the pathogens that are associated with ALRIs and mixed viral-bacterial infections. Secondly, virus (sub)typing was not performed systematically and (sub)typing data were not collected., These data are very important and could provide a more comprehensive picture by age group and seasonality in various regions [28]. Thirdly, further understanding of seasonality of these viral agents in various climate regions and co-relation with meteorological data (temperature, rainfall, humidity etc.) will be important to better understand and describe the epidemiology of these etiologies and related diseases, and for appropriately timing the use of interventions, such as influenza vaccines and future RSV vaccines [30]. Fourthly, this study did not include HRV during the first four years, which is also one of most common pathogens associated with ALRI. 5) The importance of viruses as major causes of ALRIs is becoming increasingly apparent because the sensitivity of detection techniques has greatly improved and new molecular tests are increasingly replacing conventional methods. However, lack of controls limits our ability to infer a causal association and therefore our results must be interpreted with caution [31], [32].

## Conclusion

In conclusion, this study provided important background information concerning the respiratory viral etiologies in China, based on a large sample size across a vast territory for multiple seasons. Our findings could serve as robust evidence for public health authorities for drawing up further plans to prevent and control of respiratory virus associated ALRIs. The spectrum of viral etiologies could be helpful to estimate disease burden associated with these pathogens and to guide the priority for future research studies and allocate resources to fight infectious diseases. RSV is common in very young children and prevention measures, such as vaccination, could have a large public health impact. Influenza was most common in adults and influenza vaccination should be more widespread in China. The seasonality information could be used as a basis to optimize the timing for the potential use of appropriate pharmaceutical and nonpharmaceutical interventions against these diseases. These preliminary results indicate that more robust surveillance data and evaluations are needed to estimate the disease burden and to understand whether geographic areas, climate and other environmental factors and patterns of human behavior influence the timing and severity of epidemics associated with these viral agents.

## Supporting Information

Figure S1 **Number and percentage of patients positive for ADV by age group.** A) 0–4 years. B: 5–64 years) C) ≥65 years.
(TIF)

Figure S2 **Number and percentage of patients positive for hCoV by age group.** A) 0–4 years. B: 5–64 years) C) ≥65 years.
(TIF)

File S1 **Tables S1 & S2.** Table S1. Primers and sequence information to detect viral etiologies by RT-PCR or PCR in this study. Table S2. Primers and sequence information to detect viral etiologies by Real-time RT-PCR or PCR in this study.
(DOCX)
