# Supplementary figures and images for: Viral Etiologies of Hospitalized Acute Lower Respiratory Infection Patients in China, 2009-2013
Source: PLoS One. 2014 Jun 19;9(6):e99419. doi: 10.1371/journal.pone.0099419 (PMC4063718; doi:10.1371/journal.pone.0099419)

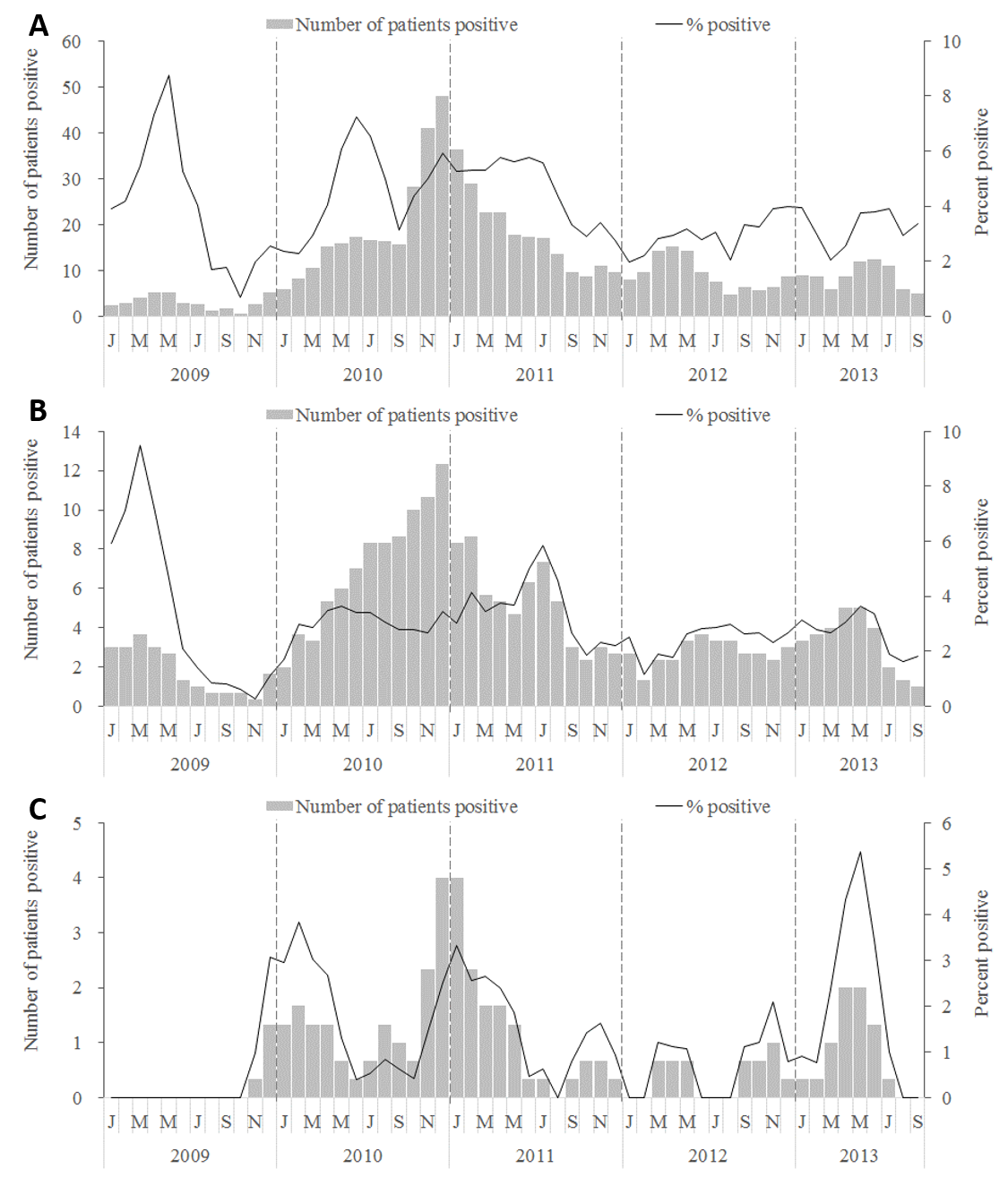

Supplement: Figure S1 — Number and percentage of patients positive for ADV by age group. A) 0–4 years. B: 5–64 years) C) ≥65 years. (TIF) [file pone.0099419.s001.tif]

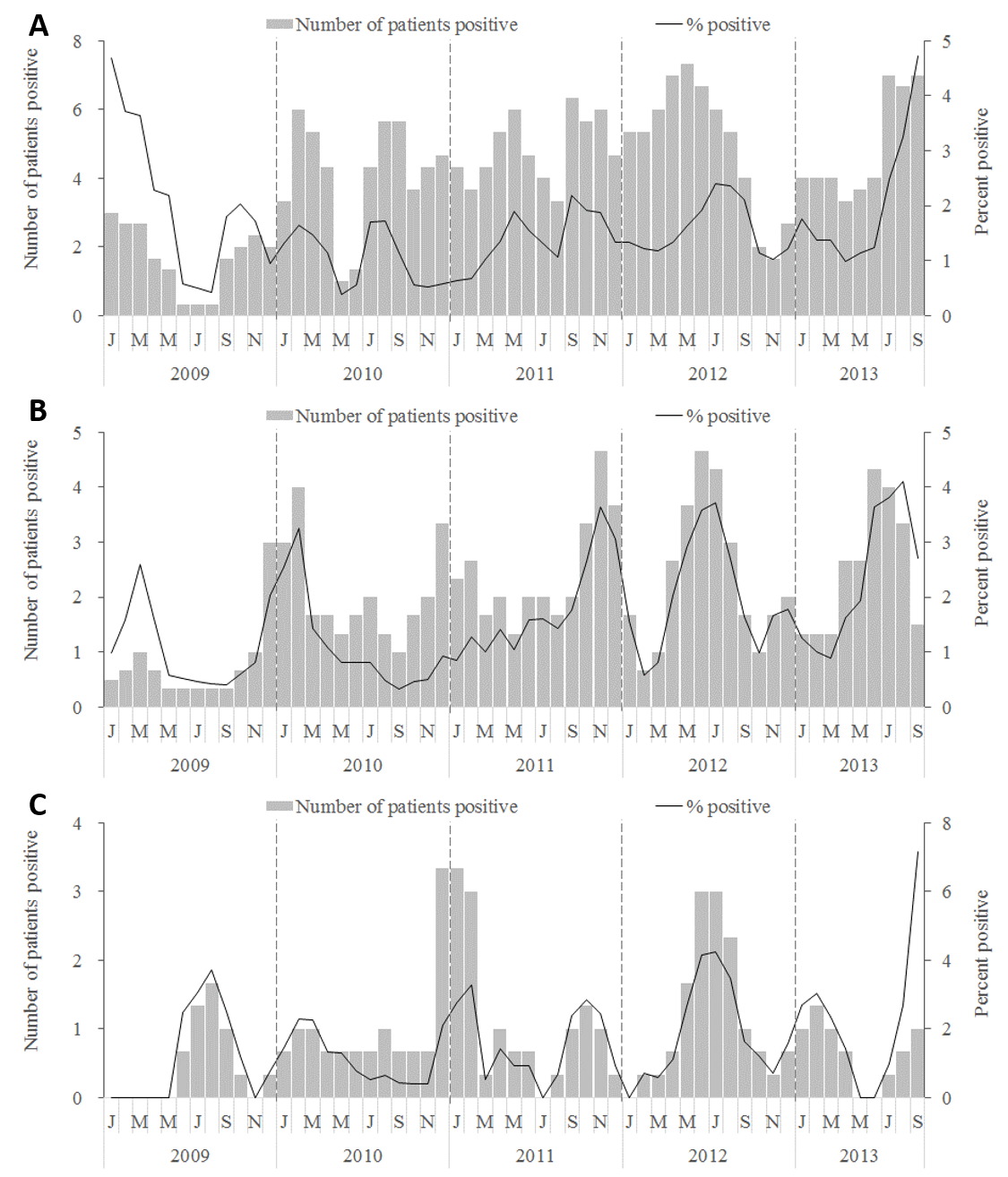

Supplement: Figure S2 — Number and percentage of patients positive for hCoV by age group. A) 0–4 years. B: 5–64 years) C) ≥65 years. (TIF) [file pone.0099419.s002.tif]
