# Supplementary material for: Viral Etiologies of Hospitalized Acute Lower Respiratory Infection Patients in China, 2009-2013
Source: PLoS One. 2014 Jun 19;9(6):e99419. doi: 10.1371/journal.pone.0099419 (PMC4063718; doi:10.1371/journal.pone.0099419)
Supplement: File S1 — Tables S1 & S2. Table S1. Primers and sequence information to detect viral etiologies by RT-PCR or PCR in this study. Table S2. Primers and sequence information to detect viral etiologies by Real-time RT-PCR or PCR in this study. (DOCX) [file pone.0099419.s003.docx]

**Viral Etiologies of Hospitalized Acute Lower Respiratory Infection Patients in China, 2009-2013**

**Supplementary Information**

Luzhao Feng^1☯^, Zhongjie Li^1☯^, Shiwen Zhao^2☯^, Harish Nair^3,4^, Shengjie Lai^1^, Wenbo Xu^5^, Mengfeng Li^6^, Jianguo Wu^7^, Lili Ren^8^, Wei Liu^9^, Zhenghong Yuan^10^, Yu Chen^11^, Xinhua Wang^12^, Zhuo Zhao^13^, Honglong Zhang^1^, Fu Li^6^, Xianfei Ye^10^, Sa Li^1^, Daniel Feikin^14^, Hongjie Yu^1^*****, Weizhong Yang^1^*****

1 Division of Infectious Disease, Key Laboratory of Surveillance and Early-warning on Infectious Disease, Chinese Centre for Disease Control and Prevention, Beijing, China

2 Yunnan Provincial Center for Disease Control and Prevention, Kunming, China

3 Centre for Population Health Sciences, Global Health Academy, The University of Edinburgh, Edinburgh, UK

4 Public Health Foundation of India, New Delhi, India

5 National Institute for Viral Disease Control and Prevention, Chinese Center for Disease Control and Prevention, Beijing, China

6 Key Laboratory of Tropical Disease Control, Ministry of Education, Sun Yat-Sen University, Guangzhou, China

7 State Key Laboratory of Virology, College of Life Sciences, Wuhan University, Wuhan, China

8 Institute of Pathogen Biology, Chinese Academy of Medical Sciences & Peking Union Medical College, Beijing, China

9 Beijing Institute of Microbiology and Epidemiology, State Key Laboratory of Pathogen and Biosecurity, Beijing, China

10 Shanghai Public Health Clinical Center, Shanghai, China

11 State Key Laboratory for Diagnosis and Treatment of Infectious Diseases, First Affiliated Hospital, School of Medicine, Zhejiang University, Hangzhou, China

12 Gansu Provincial Center for Disease Control and Prevention, Lanzhou, China

13 Liaoning Provincial Center for Disease Control and Prevention, Shenyang, China

14 Division of Viral Diseases, National Center for Immunization and Respiratory Diseases, Centers for Disease Control and Prevention, Atlanta, USA

Table S1. Primers and sequence information to detect viral etiologies by RT-PCR or PCR in this study

| Virus | Primer | Sequence (5’-3’) | Gene | Amplification  size |
| --- | --- | --- | --- | --- |
| RT-PCR |  |  |  |  |
| Influenza [1] | FluAB3 | GATCAAGTGAKMGRRAGYMGRAAYCCAGG | NP | A: 301bp  B: 226bp  C: 111bp |
|  | FluC3 | AAATTGGAATTTGTTCCTTTCAAGGGACA |  |  |
|  | FluAC4 | TCTTCAWATGCARSWSMAWKGCATGCCATC |  |  |
|  | FluB4 | CTTAATATGGAAACAGGTGTTGCCATATT |  |  |
| RSV [1] | RSVA3 | TTATACACTCAACAATRCCAAAAAWACC | F | A: 363 bp  B: 611 bp |
|  | RSVA4 | AAATTCCCTGGTAATCTCTAGTAGTAGTCTGT |  |  |
|  | RSVB3 | ATCTTCCTAACTCTTGCTRTTAATGCATTG |  |  |
|  | RSVB4 | GATGCGACAGCTCTGTTGATTTACTATG |  |  |
| PIV [2] | 1PIV13 | AGGWTGYSMRGATATAGGRAARTCAT | HA | PIV1: 439bp  PIV2: 297bp  PIV3: 390bp  PIV4: 174bp |
|  | 2PIV13 | CTWGTATATATATRTAGATCTTKTTRCCTAGT |  |  |
|  | 1PIV2 | TAATTCCTCTTAAAATTGACAGTATCGA |  |  |
|  | 1PIV4 | ATCCAGARRGACGTCACATCAACTCAT |  |  |
|  | 2PIV24 | TRAGRCCMCCATAYAMRGGAAATA |  |  |
| hMPV [3] | hMPVLF | CATGCCCACTATAAAAGGTCAG | L | 171bp |
|  | hMPVLR | CACCCCAGTCTTTCTTGAAA |  |  |
| hCoV [4] | hCoVFc | GGTTGGGACTATCCTAAGTGTGA | POL | 440bp |
|  | hCoVRc | CCATCATCAGATAGAATCATCATA |  |  |
| PCR |  |  |  |  |
| ADV [5] | 1-ADVF | GCCSCARTGGKCWTACATGCACATC | Hexon | 301bp |
|  | 1-ADVR | CAGCACSCCICGRATGTCAAA |  |  |
| hBoV [6] | HBoVF | GACCTCTGTAAGTACTATTAC | NP1 | 354bp |
|  | HBoVR | CTCTGTGTTGACTGAATACAG |  |  |

RSV: Respiratory syncytial virus; ADV: adenovirus virus; PIV: parainfluenza virus; hMPV: human metapneumovirus; hCoV: human coronavirus; hBoV: human bocavirus.

Table S2. Primers and sequence information to detect viral etiologies by Real-time RT-PCR or PCR in this study

| Virus | Primers | Sequence (5’-3’) | 3' Label | 5' Label | Gene |
| --- | --- | --- | --- | --- | --- |
| Influenza [7,8] | FluA-F  FluA-R  FluA-P | GACCRATCCTGTCACCTCTGAC  AGGGCATTYTGGACAAAKCGTCTA  TGCAGTCCTCGCTCACTGGGCACG | TAMRA | FAM | M_2_ |
|  | 09FluH1-F  09FluH1-R  09FluH1-P | GTGCTATAAACACCAGCCTYCCA  CGGGATATTCCTTAATCCTGTRGC  CAGAATATACA”T”CCRGTCACAATTGGARAA | TAMRA | FAM | HA |
|  | FluB-F  FluB-R  FluB-P | TGCCTACCTGCTTTMMYTRACA  CCRAACCAACARTGTAATTTTTCTG  TGCTTTGCCTTCTCCA | TAMRA | FAM | M |
| RSV [9] | RSVA-F  RSVA-R  RSVA-P | GCTCTTAGCAAAGTCAAGTTGAATGA  TGCTCCGTTGGATGGTGTATT  ACACTCAACAAAGATCAACTTCTGTCATCCAGC | TAMRA | FAM | N |
|  | RSVB-F  RSVB-R  RSVB-P | GATGGCTCTTAGCAAAGTCAAGTTAA  TGTCAATATTATCTCCTGTACTACGTTGAA  TGATACATTAAATAAGGATCAGCTGCTGTCATCCA | TAMRA | FAM | N |
| PIV [10] | hPIV1-F  hPIV1-R  hPIV1-P | TGATTTAAACCCGGTAATTTCTCAT  CCTTGTTCCTGCAGCTATTACAGA  ACGACAACAGGAAATC | TAMRA | FAM | HN |
|  | hPIV2-F  hPIV2-R  hPIV2-P | AGGACTATGAAAACCATTTACCTAAGTGA  AAGCAAGTCTCAGTTCAGCTAGATCA  ATCAATCGCAAAAGCTGTTCAGTCACTGCTATAC | TAMRA | FAM | HN |
|  | hPIV3-F  hPIV3-R  hPIV3-P | TGATGAAAGATCAGATTATGCATCATC  CCGGGACACCCAGTTGTG  TGGACCAGGGATATACTACAAAGGCAAAATAATATTTCTC | TAMRA | FAM | HN |
|  | hPIV4-F  hPIV4-R  hPIV4-P | CAAAYGATCCACAGCAAAGATTC  ATGTGGCCTGTAAGGAAAGCA  GTATCATCATCTGCCAAATCGGCAATTAAACA | TAMRA | FAM | Nucleoc-apsid |
| hMPV [11] | hMPV-F  hMPV-R  hMPV-P | CATAYAARCATGCTATATTAAAAGAGTCTC  CCTATYTCTGCAGCATATTTGTAATCAG  TGYAATGATGARGGTGTCACTGCRGTTG | TAMRA | FAM | NP |
| hCoV [12] | 229E-F  229E-R  229E-P | CAGTCAAATGGGCTGATGCA  AAAGGGCTATAAAGAGAATAAGGTATTCT  CCCTGACGACCACGTTGTGGTTCA | TAMRA | FAM | NP |
|  | NL63-F  NL63-R  NL63-P | GACCAAAGCACTGAATAACATTTTCC  ACCTAATAAGCCTCTTTCTCAACCC  AACACGCTTCCAACGAGGTTTCTTCAACTGAG | TAMRA | FAM | NP |
|  | OC43-F  OC43-R  OC43-P | CGATGAGGCTATTCCGACTAGGT  CCTTCCTGAGCCTTCAATATAGTAACC  TCCGCCTGGCACGGTACTCCCT | TAMRA | FAM | NP |
|  | HKU1-F  HKU1-R  HKU1-P | CCTTGCGAATGAATGTGCT  TTGCATCACCACTGCTAGTACCAC  TGTGTGGCGGTTGCTATTATGTTAAGCCTG | TAMRA | FAM | Replicase1b |
| ADV [13] | hAdV-F  hAdV-R  hAdV-P | GCCACGGTGGGGTTTCTAAACTT  GCCCCAGTGGTCTTACATGCACATC  TGCACCAGACCCGGGCTCAGGTACTCCGA | TAMRA | FAM | Hexon |
| hBoV [14] | HBoV-F  HBoV-R  HBoV-P | AGAGGCTCGGGCTCATATCA  CACTTGGTCTGAGGTCTTCGAA  AGGAACACCCAATCARCCACCTATCGTCT | TAMRA | FAM | NP-1 |

B =C, G, or T; H =A, C, or T; R= A or G; S =G or C; Y =C or T.

RSV: Respiratory syncytial virus; ADV: adenovirus virus; PIV: parainfluenza virus; hMPV: human metapneumovirus; hCoV: human coronavirus; hBoV: human bocavirus; HN: Hemagglutinin neuraminidase.

**References:**

1. Coiras MT, Pérez-Breña P, García ML, Casas I (2003) Simultaneous detection of influenza A, B, and C viruses, respiratory syncytial virus, and adenoviruses in clinical samples by multiplex reverse transcription nested- PCR assay. J Med Virol 69: 132-144.
2. Coiras MT, Aguilar JC, García ML, Casas I, Pérez-Breña P (2004) Simultaneous detection of fourteen respiratory viruses in clinical specimens by two multiplex reverse transcription nested-PCR assays. J Med Virol 72: 484-495.
3. Peiris JS, Tang WH, Chan KH, Khong PL, Guan Y, et al. (2003) Children with respiratory disease associated with metapneumovirus in Hong Kong. Emerg Infect Dis 9: 628-33.
4. Woo PC, Lau SK, Chu CM, et al. (2005) Characterization and complete genome sequence of a novel coronavirus, coronavirus HKU1, from patients with pneumonia. J Virol 79: 884-895.
5. Allard AK, Girones R, Juto P, Wadell G (1990) Polymerase chain reaction for detection of adenoviruses in stools. J Clin Microbiol 28: 2659-2667.
6. Chung JY, Han TH, Kim CK,Kim SW. (2006) Bocavirus Infection in Hospitalized Children, South Korea. Emerg Infect Dis 12(8):1254-1256.
7. WHO: CDC protocol of realtime RT-PCR for influenza A (H1N1), 30 April 2009.
8. Glenys R. Chidlow, Gerry B. Harnett, Geoffrey R. Shellam et al. (2009) An Economical Tandem Multiplex Real-Time PCR Technique for the Detection of a Comprehensive Range of Respiratory Pathogens.Viruses 1: 42-56.
9. Aizhong Hu, Melissa Colella, John S. Tam, et al. (2003) Simultaneous Detection, Subgrouping, and Quantitation of Respiratory Syncytial Virus A and B by Real-Time PCR. Journal of Clinical Microbiology, 41: 149–154.
10. Alma C. van de Pol, Anton M. van Loon, Tom F. W. Wolfs, et al. (2007) Increased Detection of Respiratory Syncytial Virus, Influenza Viruses, Parainfluenza Viruses, and Adenoviruses with Real-Time PCR in Samples from Patients with Respiratory Symptoms. Journal of Clinical Microbiology 45: 2260–2262.
11. Jeroen Maertzdorf, Chiaoyin K. Wang, Jennifer B. Brown, et al. (2004) Real-Time Reverse Transcriptase PCR Assay for Detection of Human Metapneumoviruses from All Known Genetic Lineages. Journal of Clinical Microbiology 42: 981–986.
12. Ryan K. Dare,Alicia M. Fry,Malinee Chittaganpitch, et al. (2007) Human coronavirus infections in rural Thailand: a comprehensive study using real-time reverse-transcription polymerase chain reaction assays. The Journal of Infectious Diseases 196: 1321–1328.
13. Albert Heim, Carmen Ebnet, Gabi Harste, et al. (2003) Rapid and Quantitative Detection of Human Adenovirus DNA by Real-Time PCR. Journal of Medical Virology 70: 228–239.
14. Xiaoyan Lu, Malinee Chittaganpitch, Sonja J. Olsen, et al. (2006) Real-Time PCR Assays for Detection of Bocavirus in Human Specimens. Journal of Clinical Microbiology 44: 3231–3235.
